# TNF-α Priming Elicits Robust Immunomodulatory Potential of Human Tonsil-Derived Mesenchymal Stem Cells to Alleviate Murine Colitis

**DOI:** 10.3390/biomedicines8120561

**Published:** 2020-12-02

**Authors:** Tae-Hoon Shin, Ji-Su Ahn, Su-Jeong Oh, Ye Young Shin, Ji Won Yang, Min-Jung Kang, Ji Min Kim, Byung-Joo Lee, Yoojin Seo, Hyung-Sik Kim

**Affiliations:** 1 Translational Stem Cell Biology Branch, National Heart, Lung, and Blood Institute, National Institutes of Health, Bethesda, MD 20892, USA; thshin1125@gmail.com; 2 Dental and Life Science Institute, Pusan National University, Yangsan 50612, Korea; anjs08@naver.com (J.-S.A.); dhtnwjd26@naver.com (S.-J.O.); bubu3935@naver.com (Y.Y.S.); midnightnyou@naver.com (J.W.Y.); kkang085@naver.com (M.-J.K.); 3 Department of Life Science in Dentistry, School of Dentistry, Pusan National University, Yangsan 50612, Korea; 4 Department of Otorhinolaryngology-Head and Neck Surgery, Biomedical Research Institute, Pusan National University School of Medicine, Pusan National University Hospital, Busan 49241, Korea; ny5thav@hanmail.net (J.M.K); voiceleebj@gmail.com (B.-J.L.)

**Keywords:** mesenchymal stem cell, palatine tonsil, immunomodulation, tumor necrosis factor alpha, cyclooxygenase-2, prostaglandin E_2_, colitis

## Abstract

Mesenchymal stem cells (MSCs) have been spotlighted in the field of cell therapies as a promising tool for the treatment of intractable inflammatory diseases. However, their therapeutic potency still shows a gap between preclinical and clinical settings, and distinctive characteristics of specific tissue-derived MSCs and definitive ways to maximize their beneficial functions have not been fully elucidated yet. We previously identified the unique MSCs population from human palatine tonsil (TMSCs) and revealed their superior properties in proliferation and ROS regulation. Based on these findings, we explored further characteristics of TMSCs particularly focused on immunomodulatory function. We found the merit of TMSCs as a therapeutic agent that retains favorable MSCs properties until relatively late passages and revealed that pre-treatment of TNF-α can enhance the immunomodulatory abilities of TMSCs through the upregulation of the PTGS2/PGE_2_ axis. TMSCs primed with TNF-α effectively restrained the proliferation and differentiation of T lymphocytes and macrophages in vitro, and more interestingly, these TNF-α-licensed TMSCs exhibited significant prophylactic and therapeutic efficacy in a murine model of autoimmune-mediated acute colitis via clinical and histopathological assessment compared to unprimed naïve TMSCs. These findings provide novel insight into the optimization and standardization of MSCs-based anti-inflammatory therapies, especially targeting inflammatory bowel disease (IBD).

## 1. Introduction

Mesenchymal stem cells (MSCs)-based therapy has emerged as one of the most promising alternatives for the treatment of various intractable diseases taking advantage of several mechanisms such as regeneration, homing, immunomodulation and resident cell activation [1,2]. Indeed, authentic properties as a stem cell including self-renewal and differentiation enabled MSCs to be extensively utilized in the field of regenerative medicine such as wound healing [3] and repair of bone and cartilage defects [4,5]. In addition, MSCs have been revealed to exhibit low immunogenicity and exert immunomodulatory function by actively interacting with both innate and adaptive immune cells [6,7], most likely through the secretion of regulatory mediators including indoleamine 2,3-dioxygenase (IDO), nitric oxide (NO), prostaglandin E2 (PGE_2_), transforming growth factor beta (TGF-β), and interleukin 10 (IL-10) [8,9,10,11]. Based on this unique feature, accumulating evidence has reported the safety and efficacy of MSCs through the regulation of host immune microenvironment in autoimmune diseases (e.g., rheumatoid arthritis [12], diabetes, [13] and inflammatory bowel disease [14]), allergic disorders (e.g., rhinitis [15], asthma [16] and atopic dermatitis [17]), and even recently, infectious diseases like COVID-19 [18]. However, despite the promising outcomes in early experimental and clinical settings, MSCs-based therapeutics have shown only limited potency, especially in advanced trials. Therefore, there have been strong needs for advanced approaches to enhance MSCs function and efficacy along with intensive studies on the unique characterization of MSCs derived from specific tissues and standardization of therapy protocol.

Almost all adult tissues (e.g., bone marrow, adipose tissue, liver, skin, tonsil and gingiva) and some perinatal tissues (e.g., amnion, umbilical cord tissue, cord blood, and placenta) can be utilized as a source for MSCs acquisition [19]. Although MSCs populations derived from different tissue sources generally meet common criteria including self-renewal, differentiation and surface marker expression, MSCs themselves are heterogenous even within the tissue and may have different characteristics and functions depending on their origin [19,20]. Therefore, to accomplish adequate outcomes, it should be preceded to identify the distinctive characteristics among MSCs from different sources and select the appropriate MSCs for the target disease. Moreover, a series of studies have suggested that the strategic approaches prior to administration, such as pre-conditioning, genetic manipulation, and modification of the cell culture environment, can compensate for the limited efficacy of naïve MSCs therapy, which indicates that even a limited number of MSCs and doses could lead to satisfactory results [21]. Among them, pre-conditioning with specific biological factors, such as cytokines, growth factors, and ligands, has been considered the easiest and effective way to improve MSCs function. However, persistent studies on the unique function of specific tissue-derived MSCs, especially those from recently reported novel tissues, should be required to develop optimal methods for functional enhancement.

Palatine tonsils are one of the novel promising tissue sources of MSCs because they can be easily obtained via surgical tonsillectomy with minimally invasive procedures. Since the procedure is usually conducted on younger patients between 5 to 19 than the elderly, tonsil-derived MSCs (TMSCs) are relatively free from concerns related to donor ageing, which is demonstrated by higher yields and faster growth rates than MSCs derived from the same amount of bone marrow and adipose tissue [22]. TMSCs have been revealed to possess common features of MSCs including the multipotent differentiation capability and immunomodulation, and extensive investigations into their therapeutic applicability and underlying mechanisms, as in other types of MSCs, are actively underway [23]. We previously identified that a distinctive TMSCs population expressing a novel stem cell marker, W5C5, reside in the perivascular regions of the palatine tonsil [24], and that higher expression of fibroblast growth factor 5 (FGF-5) in TMSCs may be a key mechanism of the superior proliferative potential compared to MSCs obtained from bone marrow and adipose tissue [25]. In addition, we described the beneficial effects of TMSCs on wound healing process predominantly through anti-inflammatory and anti-fibrotic mechanisms of action [3], and more recently, demonstrated the essential role of stanniocalcin-1 (STC1) overexpressed in TMSCs in proliferation and defense mechanisms against oxidative stress [26]. Other groups have also documented the therapeutic potential of TMSCs in various disorders including allergic rhinitis [27], hepatic fibrosis [28] and peripheral nerve injury [29]. However, much still remains uncovered about TMSCs in terms of precise identification and molecular mechanisms responsible for specific functions. Furthermore, few studies have demonstrated functional enhancement strategies in TMSCs-based therapy.

In this study, we sought to demonstrate whether licensing of TMSCs with proinflammatory cytokines, interferon gamma (IFN-γ) and/or tumor necrosis factor alpha (TNF-α), impacts their immunogenicity and immunosuppressive effects particularly on T cells and macrophages, as well as to verify the remarkable prophylactic and therapeutic potency in dextran sulfate sodium (DSS)-induced murine acute colitis model compared to naïve TMSCs.

## 2. Materials and Methods

### 2.1. TMSCs Culture and Cytokine Pre-Treatment

All the procedures performed in this study were approved by the Institutional Review Board of Pusan National University Hospital (IRB No. H-1801-033-062, 22/01/2018) and written informed consent was obtained from all patients or their parents. TMSCs were isolated and cultured as described previously [26]. Briefly, the palatine tonsils excised during surgical tonsillectomy were washed with sterile phosphate-buffered saline (PBS), followed by the enzymatic digestion with 0.075% type I collagenase (Sigma-Aldrich, St. Louis, MO, USA) at 37 °C for 30 min. The prepared single cell suspension was filtered through a 100 µm nylon mesh and incubated overnight in α-minimum essential media (α-MEM, Gibco, Grand Island, NY, USA) supplemented with 10% fetal bovine serum (FBS, Gibco) at 37 °C with 5% CO_2_. After washing with PBS, the adherent TMSCs population was maintained and expanded for experimentations. TMSCs between passage 5 and 7 obtained from five different donors were used for this study.

TMSCs were primed with 20 ng/mL of TNF-α (Peprotech, Rocky Hill, NJ, USA) and/or 20 ng/mL of IFN-γ (Peprotech) for 24 h prior to subsequent experiments.

### 2.2. Cell Proliferation

To assess the TMSCs proliferation capability and cellular senescence, cumulative population doubling level (CPDL) was calculated based on the formula:

CPDL = ln (Nf/Ni) ln2

Ni and Nf stand for the initial and final number of cells counted, respectively. TMSCs were plated into a 6-well plate at a density of 6 × 10^4^ cells/well and cultured in α-MEM media containing 10% FBS. The cells were subcultured every 3 days and the number of viable cells was quantified at each subculture. The population level calculated at each passage was tracked and cumulated up to 12 passages from primary culture.

### 2.3. Osteogenic and Adipogenic Differentiation

To assess the differentiation capability into osteogenic and adipogenic lineages, TMSCs were plated in 6-well plates at a confluency of 1.5 × 10^5^ cells/well in the cell culture media. When the cells reach about 40 to 50% of confluency, media was changed to osteogenic differentiation media, low glucose Dulbecco’s Modified Eagle Medium (DMEM) media (Gibco) containing 0.1 µM dexamethasone, 10 mM beta-glycerophosphate, 50 µM ascorbate (Sigma-Aldrich), and 10% FBS, and was replaced every 2 or 3 days. After 2 weeks of differentiation, cells were washed with PBS and fixed with 70% EtOH at 4 °C for 20 min. The fixed TMSCs were stained with 1 mL of Alizarin Red S (Kanto, Tokyo, Japan) at room temperature for 10 min, and the stained cells were pictured by optical microscopy following washing twice with distilled water.

The induction of adipogenic differentiation was initiated when the cell density reaches up to 80% by replacing media with adipogenic differentiation media consisted of 10% FBS, 1 M dexamethasone, 0.5 mM 3-isobutyl-1-methylxanthine, 0.2 mM indomethacin, and 10 µg/mL insulin (Sigma-Aldrich). The media was replaced every 3 days and at 2 weeks post-differentiation, TMSCs were fixed with 70% EtOH and stained with 1 mL of Oil Red O (Sigma-Aldrich) working solution at room temperature for 1 h. Representative fields of the stained wells were captured using optical microscopy.

### 2.4. Cell Surface Marker Expression

The expression of cell surface markers for TMSCs was determined by flow cytometric analysis. TMSCs were harvested and resuspended in 50 µL of staining buffer, PBS containing 5% FBS, per million cells. Antibodies specific for human CD14, CD34, C44, CD73, CD90, HLA-DR, and HLA-ABC were added to the cell suspension at a ratio of 1:100 and incubated at 4 °C for 1 h avoided from the direct light. Non-specific isotype antibodies were used as a control. After washing with PBS, cells were resuspended in 500 µL of staining buffer containing 0.4% paraformaldehyde and the fixed samples were analyzed by FACSCalibur (BD Biosciences, Franklin Lakes, NJ, USA) within 24 h. All antibodies stained were purchased from BD Biosciences and the acquired results were analyzed using FlowJo 10 software (FlowJo, LLC, Ashland, OR, USA).

### 2.5. Quantitative Real-Time PCR (qRT-PCR)

Total RNA was extracted with Trizol Reagent (Invitrogen, Carlsbad, CA, USA) from TMSCs primed with IFN-γ, TNF-α or both as well as naïve controls according to the manufacturer’s instruction. cDNA was reversely transcribed from 1 µg of the extracted RNA using ReverTra Ace^®^ qPCR RT Master Mix (Toyobo, Osaka, Japan), and the expression of *PTGS2* (cyclooxygenase 2, *COX-2*) gene was determined by SYBR^®^ Green-based quantitative PCR method using an ABI 7500 real-time PCR system (Applied Biosystems, Foster City, CA, USA). Relative mRNA expression of the target genes was quantified by the comparative Ct method and normalized by that of the housekeeping gene, *GAPDH* (glyceraldehyde 3-phosphate dehydrogenase). Primer sequences used in this study include: *PTGS2* forward: TGAGCATCTACGGTTTGCTG, reverse: TGCTTGTCTGGAACAACTGC; *GAPDH* forward: GTCTCCTCTGACTTCAACAGCG, reverse: ACCACCCTGTTGCTGTAGCCAA.

### 2.6. Mixed Lymphocyte Reaction

TMSCs with or without cytokine priming were treated with 25 mg/mL of mitomycin C (Sigma-Aldrich) at 37 °C for 1 h to hinder cell proliferation, followed by seeding into 96-well plates at a density of 1x10^4^ cells/well. Peripheral blood mononuclear cells (PBMCs, Zenbio, Research Triangle Park, NC, USA) were added to TMSCs-plated well for coculture in RPMI1640 media (Gibco) containing 10% FBS in the presence of concanavalin A (ConA 5 µg/mL, Sigma-Aldrich) or anti-CD3 (5 µg/mL)/anti-CD28 (2 µg/mL, eBioscience, San Diego, CA, USA) for the activation of pan-leukocytes or T lymphocytes, respectively. The proliferation of PBMCs or T lymphocytes was determined using Cell Proliferation ELISA, bromodeoxyuridine (BrdU) Kit (Roche, Indianapolis, IN, USA) following 5 days of coculture.

To assess the immunogenicity of TMSCs, naïve and primed TMSCs were cocultured with the PBMCs (TMSCs:PBMCs = 1:10) without any stimuli, and the PBMC proliferation was measured compared with the results from PBMCs treated with mitogen or immune stimulants such as ConA and anti-CD3/28. To evaluate the immunosuppressive effects, PBMCs were added to TMSCs at the ratio of 1:10 (TMSCs:PBMCs) under stimulation by ConA or anti-CD3/28 plus IL-2 (Peprotech). After 5 days of coculture, cell proliferation was measured by BrdU-incorporated colorimetric assay.

### 2.7. In Vitro Immune Cell Differentiation

#### 2.7.1. T Cell Differentiation

CD4^+^ helper T (Th) cells were isolated from PBMCs by magnetic-activated cell sorting (MACS) method using CD4+ T cell Isolation Kit (Miltenyi Biotec, Bergisch Gladbach, Germany) and the purified Th cells (Th0) were maintained in T cell culture media, RPMI1640 containing 25 mM HEPES, 2 mM GlutaMAX, 50 mM β-mercaptoethanol, 10% FBS and 100 U/mL penicillin/streptomycin (Gibco).

For in vitro differentiation, during Th0 cells were activated by anti-CD3 and anti-CD28 beads, IL-12 (10 ng/mL, Peprotech) and anti-IL-4 monoclonal antibody (5 µg/mL, Peprotech) were added for Th1, and IL-4 (20 ng/mL, Peprotech) and anti-IFN-γ were added for Th2 polarization. Regulatory T (Treg) cells were induced by adding TGF-β (2 ng/mL, eBioscience) and IL-2 (5 µg/mL, Peprotech) to anti-CD3/CD28. The differentiation lasted for 5 days and media was added once on day 3. After 5 days of differentiation in the presence or absence of TMSCs, cell culture supernatant was harvested and measured the concentration of IFN-γ, IL-4 and IL-10 using commercial ELISA kits (R&D Systems, Minneapolis, MN, USA) to assess the extent of Th1, Th2 and Treg cell differentiation, respectively.

TMSCs were plated into 12-well plate and primed with IFN-γ or TNF-α. After washing with PBS, Th0 cells were added to each well at a ratio of 1:10 (TMSCs:T cells) and induced differentiation into Th1 or Th2 subtype for 5 days. Th0 cells were cocultured with TMSCs at the same ratio for 5 days in the absence of any induction signals, and pre-differentiated Treg cells were used as a positive control group for IL-10 measurement.

#### 2.7.2. THP-l-Derived Macrophage-Like Cell Differentiation

THP-1 cells, a human monocytic cell line, were obtained from the Korean Cell Line Bank (Seoul, Korea). To differentiate THP-1 cells into macrophage-like cells, a million cells per well were seeded at 6-well plate in RPMI1640 media containing 10% FBS and treated with phorbol 12-myristate 13-acetate (PMA, 50 ng/mL, Sigma-Aldrich) for 48 h. After washing twice with PBS, cells were stabilized in fresh RPMI1640 media for additional 48 h. To induce M1 polarization, cells were cultured in the presence of 1 µg/mL lipopolysaccharide (LPS, InvivoGen, San Diego, CA, USA) and 20 ng/mL IFN-γ for 5 days. On day 4 of the polarization, THP-1-derived macrophage-like cells were cocultured with naïve or primed TMSCs at the ratio of 1:10 (TMSCs:THP-1) for 24 h and the concentration of TNF-α in the supernatant was measured using ELISA. To assess the impact of TMSCs on M2 polarization, differentiated macrophage-like cells right after stabilization (M0 cells) were directly cocultured with naïve or primed TMSCs for 72 h, and IL-10 level in the conditioned media was determined using ELISA.

### 2.8. Experimental Murine Colitis Induction and TMSCs Administration

All animal experiments were executed according to the regulations approved by the IACUC (PNU-2018-2034, Pusan National University). 8–10 weeks old male C57BL/6J mice were purchased from Jackson Laboratory (Bar Harbor, ME, USA). To induce experimental colitis in mice, 3% (*w*/*v*) DSS (MP Biochemicals, Solon, OH, USA) was dissolved in drinking water to keep the mice drinking for a week. 2 × 10^6^ of TMSCs with or without cytokine priming resuspended in total 200 µL of PBS were injected intraperitoneally on day 1 or day 5 of the colitis induction to investigate the prophylactic or therapeutic effects, respectively. Survival rate and body weight were constantly monitored every day and on 11 or 12 days after DSS administration, all mice were sacrificed and disease severity, colon length and histopathological lesions were evaluated.

Clinical severity of colitis was evaluated on the basis of four indices including stool consistency, rectal bleeding, coat roughness and general activity. All indices were scored in a double-blind manner from 0 to 5 according to the severity, and the sum of all scores was represented as the disease activity index (DAI). The colon resected from each mouse was measured in length from right after the cecum, and the colon tissue at a certain area was fixed and processed through the general paraffin-embedded sample process method, followed by staining with H&E for histopathological assessment. At least 4 different fields were captured from each colon using optical microscopy, and the histologic score was calculated by summing all the scores for the degree of the entire epithelium destruction, the severity of submucosal edema, and the extent of inflammatory cell infiltration in the lamina propria and submucosa.

### 2.9. Statistical Analysis

All data were represented as mean ± S.D. derived from at least three independent replicates. All statistical comparisons were analyzed by Student’s t-test or one-way ANOVA followed by the Bonferroni post hoc test for multigroup comparisons using GraphPad Prism software version 5.01 (GraphPad Software, San Diego, CA, USA). Statistical significances were marked as asterisks in each figure, and the reference group and the basis for comparison were displayed in plus sign if required.

## 3. Results

### 3.1. TMSCs Possess Genuine MSCs Features and Retain Them until Relatively Late Passages

**Figure 1:**
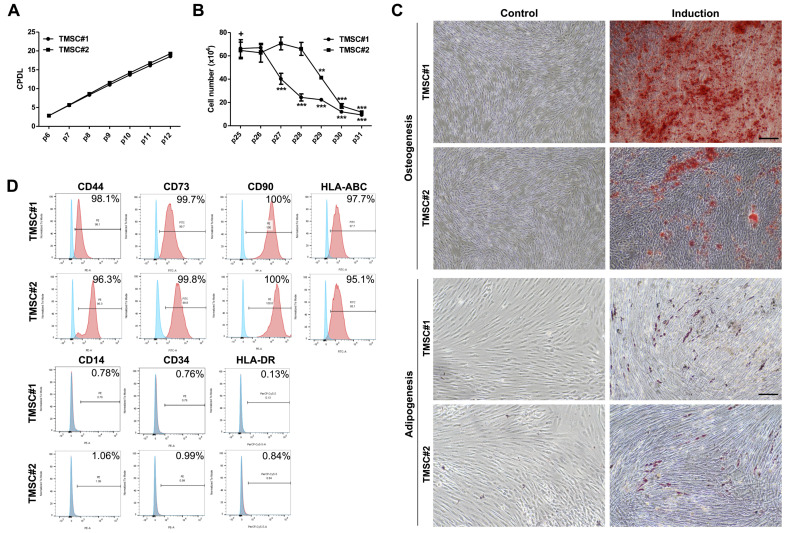
Characteristics of TMSCs as a source for MSCs therapy. (**A**) TMSCs were obtained from palatine tonsils of two different donors and the cumulative population doubling level (CPDL) was calculated through continuous passaging from p6 to p12; (**B**) To verify the onset time of cellular senescence, the absolute number of TMSCs was counted from passage 25 to 31. All data are represented as mean ± S.D. *p*-value = ** < 0.01, *** < 0.001, compared to the passage 25 marked as ‘+’; (**C**) After 2 weeks of induction into the osteogenic or adipogenic lineage, the differentiated TMSCs, as well as naïve controls, were stained with Alizarin Red S for detecting osteogenesis (**left**) and Oil Red O stain (**right**) for adipogenesis. Representative microscopic images were shown, scale bar = 250 µm; (**D**) The expression of typical positive (**top**) and negative (**bottom**) MSCs surface markers was determined by flow cytometric analysis. The peak of TMSCs stained with isotype IgG control was shown in blue and those with a specific antibody in red in the histogram.

We first investigated the characteristics of TMSCs to ensure that the cells meet the principal criteria for MSCs suitable as therapeutics. The proliferation of TMSCs obtained from two different donors was determined during the successive passaging. Both TMSCs batches showed sustainable proliferation capability up to 12 passages from the primary culture in CPDL analysis (Figure 1A). Since repeated serial passaging leads MSCs to replicative senescence associated with functional impairment, we counted the absolute number of TMSCs in the late passages after 25 where cellular senescence is expected to progress. While the proliferation of TMSC#2 began to decrease from p27, it was maintained to some extent up to p29 in TMSC#1 (Figure 1B). These findings indicate that TMSCs possess an outstanding proliferative potential that may be superior to MSCs obtained from conventional tissue sources such as bone marrow, as reported previously [22], and that the timing of the onset of TMSCs aging is relatively late in terms of self-renewal and proliferation. To assess the multilineage differentiation potential, TMSCs were cultured in differentiation induction media specific for adipogenesis or osteogenesis. After two weeks of the induction, the cells were stained with either Alizarin Red S to detect calcium deposition by osteogenesis or Oil Red O to visualize lipid droplet accumulation by adipogenesis. All TMSCs were efficiently differentiated into both adipocytes and osteoblasts. Compared to undifferentiated control TMSCs, differentiated cells exhibited obvious morphologic changes and distinct positivity to the specific staining (Figure 1C). We next examined the expression of cell surface markers to evaluate the immunophenotype of TMSCs. Flow cytometric analysis revealed that TMSCs expressed the typical MSCs markers such as CD44, CD73, CD90, and HLA-ABC whereas lacked expression of CD14, CD34, and HLA-DR (Figure 1D). These results suggest that our TMSCs possess genuine MSCs properties and retain the proliferative capability until relatively late passages, which can be advantageous for therapeutic application.

### 3.2. TNF-α Pretreatment Remarkably Augments Cyclooxygenase-2 (COX-2)/PGE_2_ Pathway in TMSCs

**Figure 2:**
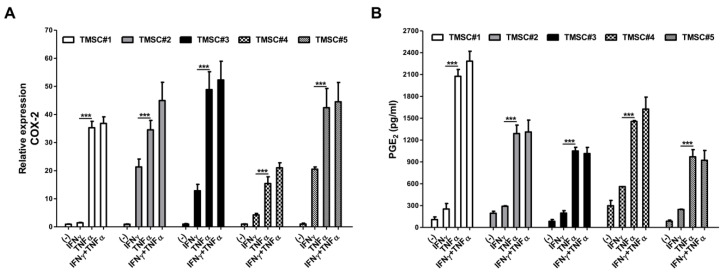
Upregulation of critical immunomodulatory factors in TMSCs by TNF-α priming. TMSCs obtained from five different individuals were primed with 20 ng/mL of IFN-γ or TNF-α or both for 24 h, and (**A**) the level of *COX-2* mRNA expression and (**B**) the secretion of PGE_2_ in cell culture supernatants were determined by real-time RT-PCR and ELISA, respectively, compared to the naïve TMSCs. All data are shown as mean ± S.D. *p*-value = *** < 0.001.

COX-2 enzymes encoded by the *PTGS2* (prostaglandin-endoperoxide synthase 2) gene catalyzes the conversion of arachidonic acid into bioreactive lipid mediator PGE_2_ which attributes to numerous pathophysiological processes such as inflammation and cancer [30]. The COX-2/PGE_2_ pathway is also known to be implicated in MSCs functions, and particularly, this axis has been considered as one of the most critical soluble mediators to exert the immunomodulatory function of MSCs among a variety of factors produced by MSCs [31]. Moreover, preliminary activation of MSCs by certain cytokines present in the inflammatory milieu triggers the activation of the axis [32]. We thus sought to determine the relative mRNA expression of *PTGS2* (*COX-2*) in TMSCs isolated from five different individuals using the real-time qPCR method as well as measured the concentration of PGE_2_ secreted into the cell culture supernatant via ELISA. To demonstrate whether pre-treatment of inflammatory cytokines could enhance the production of COX-2 and PGE_2_ in TMSCs, we primed TMSCs with IFN-γ or TNF-α or both and measured the expression of *COX-2* and the production of PGE_2_ compared with naïve TMSCs. A total of five TMSCs from different donors consistently showed the upregulation of COX-2 expression (Figure 2A) and higher production of PGE_2_ (Figure 2B) upon TNF-α pre-treatment. TMSCs primed with IFN-γ and TNF-α simultaneously also exhibited higher *COX-2* expression, but no statistical significance was found compared to those pretreated with single TNF-α. IFN-γ alone did not induce a sufficient increase in the production of both mediators. These findings suggest the potential evidence that TNF-α pre-treatment can strongly enhance the immunomodulatory function of TMSCs through upregulation of the COX-2/PGE_2_ axis, and based on these results, TMSC#1 and #2, which showed high responsiveness to TNF-α, were selected as the main cell sources for further experimentations.

### 3.3. TMSCs Exhibit the Limited Immunogenicity but Exert the Significant Suppressive Effects on Immune Cell Proliferation after TNF-α Pre-Treatment

**Figure 3:**
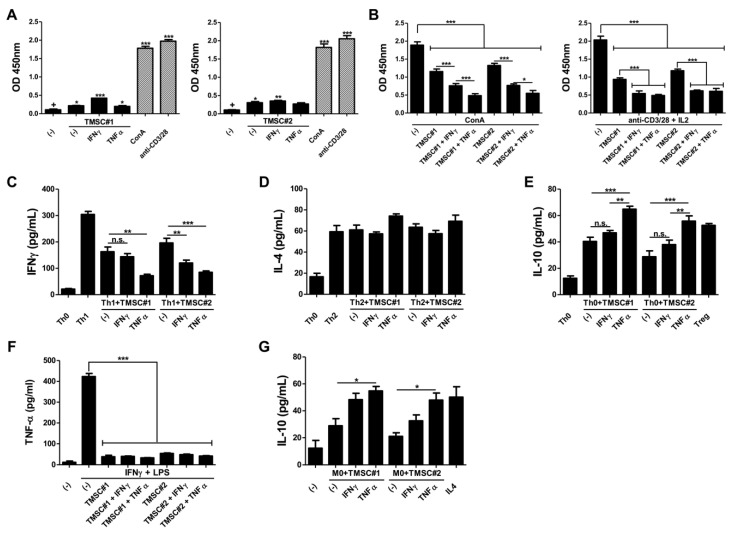
Enhanced immunomodulatory potency of TNF-α-primed TMSCs. TMSCs isolated from two different individuals were cultured for 24 h in the absence or presence of either IFN-γ or TNF-α. (**A**) Naïve or primed TMSCs were cocultured with PBMCs without any stimulants to assess the immunogenicity and proliferation of PBMCs was determined using BrdU cell proliferation ELISA kit, compared to ConA- or anti-CD3/28-treated PBMCs as positive controls; (**B**) Naïve or primed TMSCs were cocultured with PBMCs in the presence of ConA or anti-CD3/28 plus IL-2 and the suppressive effects on the immune cell proliferation were determine; (**C**,**D**) Naïve or primed TMSCs were added in the process of Th cell differentiation into Th1 or Th2 subtype and production of typical cytokines, (**C**) IFN-γ or (**D**) IL-4, was measured in the cell culture supernatant; (**E**) Treg cell induction capability of naïve and primed TMSCs was evaluated through quantifying the IL-10 concentration in the coculture supernatant; (**F**,**G**) To assess the regulatory effects on macrophage polarization, naïve or primed TMSCs were cocultured with THP-1-derived macrophages; (**F**) Primed or unprimed control TMSCs were cocultured with classically activated M1 type macrophages and the secretion of TNF-α was measured in the conditioned media; (**G**) Naïve or primed TMSCs were cocultured with resting M0 macrophages for 2 days and the production of IL-10 was determined using ELISA to assess the anti-inflammatory M2 type macrophage induction potential. All data are represented as mean ± S.D. *p*-value = * < 0.05, ** < 0.01, *** < 0.001, compared to the control group marked as ‘+’.

We next investigated whether the activated COX-2/PGE_2_ axis affects the immunomodulatory properties of TMSCs. Prior to interrogate the interactions between immune cells and TMSCs with or without cytokine, we examined the immunogenicity of naïve TMSCs versus those primed with IFN-γ or TNF-α through a coculture experiment with unstimulated PBMCs. Compared to the positive control PBMCs stimulated by either ConA or anti-CD3/CD28 beads, the proliferation of PBMCs was moderately boosted in the presence of TMSCs. Interestingly, TNF-α-primed TMSCs showed a similar extent of immunogenicity to those without pre-treatment, whereas TMSCs licensed with IFN-γ induced more a pronounced proliferation of PBMCs in both TMSCs batches analyzed (Figure 3A). Our findings propose that TMSCs have slight immunogenicity to provoke immune responses and pre-treatment of inflammatory cytokines may augment their immunogenicity.

To evaluate the suppressive effects on PBMC proliferation, naïve or primed TMSCs were cocultured with PBMCs in the presence of ConA or anti-CD3/28 plus IL-2. TMSCs themselves effectively inhibited the certain mitogen-induced immune cell proliferation, and pre-treatment of inflammatory cytokines significantly improved the inhibitory effects of TMSCs. In particular, TNF-α-primed TMSCs exhibited much higher suppression than IFN-γ-pretreated TMSCs against the proliferation of ConA-activated PBMCs. On the other hand, in the presence of T cell-specific proliferative stimuli, both cytokines showed a similar degree of significant suppressive effects, and no significant difference was observed between the two (Figure 3B). These results support that TNF-α-mediated COX-2/PGE_2_ can intensify the immunosuppressive potential in TMSCs.

### 3.4. TMSCs Activated by TNF-α Repress the Th1 Cell Differentiation and Induce Anti-Inflammatory M2 Type Macrophages

We then explored the impact of the cytokine priming on the differentiation and polarization of specific immune cell types. To assess the inhibitory effect on Th cell differentiation into certain subtypes, primed or naïve TMSCs were cocultured with purified CD4^+^ Th cells undergoing differentiation into Th1 or Th2 subtype, and the secretion of IFN-γ and IL-4 were measured for Th1 and Th2 differentiation, respectively. Terminally differentiated Th1 cells showed higher production of IFN-γ than undifferentiated Th0 cells, and TMSCs inhibited the Th1 differentiation and IFN-γ secretion. Interestingly, this suppressive effect was enhanced by cytokine pre-treatment. Particularly, TNF-α priming exhibited the most dramatic reduction in IFN-γ production both in TMSC#1 and #2, whereas IFN-γ showed an augmented inhibitory effect in only one batch of TMSCs (Figure 3C). In contrast, TMSCs did not affect Th2 differentiation. Although there was no statistical significance, TNF-α pre-treatment had an enhanced propensity to promote Th2 differentiation in both TMSCs analyzed (Figure 3D). Given that induction of suppressive cell generation, such as regulatory T (Treg) cells, is one of the primary strategies of MSCs immunomodulation, we verified the impact of TMSCs and their priming on regulatory T (Treg) cell generation. Resting Th0 cells were cocultured with either naïve or primed TMSCs without any differentiation signals and the production of IL-10 was measured in the conditioned media. Compared to the positive control that was forcibly differentiated into Treg cells, only TMSCs pretreated with TNF-α efficiently induced Treg generation and showed higher secretion of IL-10 (Figure 3E). To further examine the immunomodulatory potential of TMSCs upon TNF-α or IFN-γ priming in other immune cell types than T lymphocytes, naïve or primed TMSCs were cocultured with THP-1-derived macrophage-like cells. Regardless of TMSCs donors and priming regimen, all groups of coculture remarkably abrogated the secretion of TNF-α from activated macrophage-like cells (Figure 3F). TMSCs were cocultured with the macrophage-like cells in the resting state (M0) to assess their regulatory function on macrophages. TMSCs induced M0 cells toward anti-inflammatory M2 type cells and TNF-α pre-treatment enhanced the M2-induction capability most significantly (Figure 3G). Taken together, these findings suggest that TNF-α pre-treatment can intensify the immunomodulatory function of TMSCs, conceivably through upregulated COX-2/PGE_2_-mediated suppression of Th cell differentiation and macrophage polarization.

### 3.5. TNF-α-Primed TMSCs Exert both Prophylactic and Therapeutic Potency against DSS-Induced Murine Colitis

**Figure 4:**
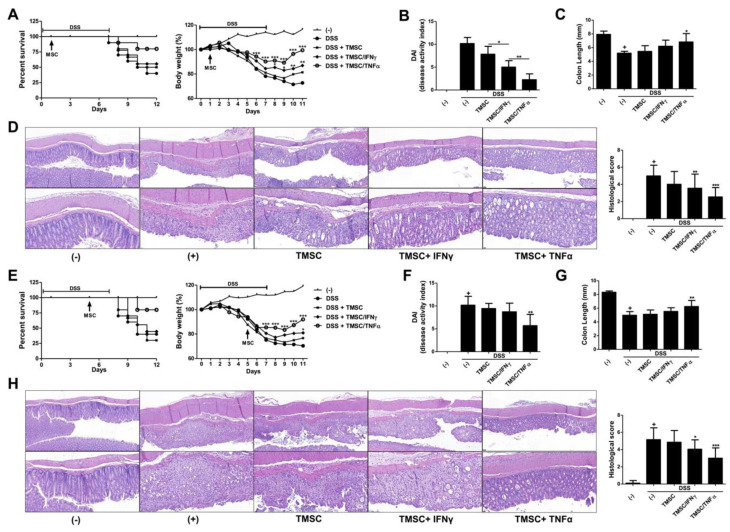
In vivo anti-inflammatory effects of TNF-α-primed TMSCs against colitis. DSS was used to induce acute colitis in C57BL/6 mice. TMSCs with or without pre-treatment were injected into colitic mice on either day 1 or day 5 of DSS induction to assess (**A–D**) preventive or (**E–H**) therapeutic potency (*n* = 5 for non-induced healthy control marked as (-), *n* = 10 for each experimental group); (**A**) Survival rate and body weight loss were consistently monitored until day 12; (**B**) Disease activity index (DAI) was determined on day 10 post colitis induction; (**C**) Gross colon length was measured after sacrifice on day 12; (**D**) Colon tissues were processed and stained with H&E for histopathologic examination. The histological score was calculated by determining the entire epithelium destruction, the submucosal edema, and the inflammatory cell infiltration in the lamina propria and submucosa, scale bar = 50 µm (**top**), 20 µm (**bottom**); (**E–H**) The same methodologies were conducted for therapeutic potency assessment; All results are shown as mean ± S.D. *p*-value = * < 0.05, ** < 0.01, *** < 0.001, compared to the control group marked as ‘+’.

To demonstrate whether IFN-γ or TNF-α priming can grant TMSCs beneficial preventive and therapeutic potency in vivo, we utilized a DSS-induced murine colitis model that T cell- and macrophage-mediated immune responses play a critical role in the disease pathogenesis. Prior to applying TMSCs directly to mice, we verified the anti-inflammatory effects of TMSCs on murine cells through coculture experiments with mouse splenocytes. Consistent with the findings from human immune cells, TMSCs pretreated with TNF-α showed the most significant suppressive effects on the mouse splenocyte proliferation (Figure S1). 3% DSS was given to mice in drinking water ad libitum, and typical symptoms of acute colitis occurred within one week of administration. TMSC#1 revealed to possess the best immunomodulation efficacy via in vitro evaluations was selected for in vivo administration, and based on the results of our previous studies, 2 × 10^6^ cells showing the highest dose-dependent efficacy of MSCs were determined as an injection dose for the mice with colitis. Intraperitoneal injection of TMSCs at day 1 of induction prevented mice from severe body weight loss and mortality. Notably, primed TMSCs exhibited more pronounced protective effects than naïve TMSCs (Figure 4A). The colitis mice as well as healthy controls were sacrificed on day 12 of induction to evaluate gross and histopathological lesions. TMSCs administration alleviated the clinical severity of colitis, with further improvement in the order of IFN-γ and TNF-α priming prior to injection (Figure 4B). Colon length measured immediately after autopsy demonstrated an overall recovery pattern by TMSCs administration, although only the TNF-α-primed group showed statistical significance (Figure 4C). Upon histopathologic assessment after H&E stain, mice given TMSCs had relatively intact colon tissues, with the highest degree in TNF-α-primed TMSCs, compared to the colon of DSS-induced control mice in terms of the entire epithelium destruction, the submucosal edema, and the inflammatory cell infiltration in the lamina propria and submucosa (Figure 4D). To assess the therapeutic potency, TMSC#1 was administered on day five post DSS induction, where acute colitis has progressed to some extent. In this approach, only TMSCs primed by TNF-α exhibited statistically significant efficacies in most examinations, including bodyweight recovery (Figure 4E), disease activity index (Figure 4F), colon length (Figure 4G), and colon histopathology (Figure 4H). Our findings indicate that TMSCs can exert protective and therapeutic potency against acute colitis in vivo, in line with the immunosuppressive capabilities verified in vitro, and furthermore, the pre-treatment of TNF-α can maximize the beneficial efficacy of TMSCs.

## 4. Discussion

Given that the anti-inflammatory actions of MSCs are exerted in response to the host microenvironment, pre-treatment with proinflammatory cytokines can be the best way to enhance the immunomodulatory function of MSCs by practicing MSCs in the mimicked in vivo inflammatory milieu in advance [33]. To date, a number of studies have reported improvements in the immune-related function of MSCs following pre-treatment with certain cytokines. IFN-γ priming was demonstrated to enhance the immunosuppressive effects of human bone marrow-derived MSCs (BM-MSCs) mainly through the upregulation of IDO [34]. While one study showed that IFN-γ-primed adipose tissue-derived MSCs (AT-MSCs) exhibited further alleviation against experimental obliterative bronchiolitis [35], another literature suggested that BM-MSCs primed by IFN-γ alone was insufficient to produce immunosuppressive effects [10]. It has been also reported that pre-treatment with TNF-α could promote the anti-inflammatory effect of MSCs via increased secretion of IL-6 and IL-8, but on the contrary, one study claimed that TNF-α-driven enhancement represented a much weaker degree than IFN-γ [36]. These contradictory results led researchers to try other pretreatment regimens like a combination of multiple cytokines [37]. In the present study, we verified that TNF-α pretreatment strengthened the immunomodulatory function of MSCs, for the first time in TMSCs from our knowledge, presumably due to the upregulation of crucial COX-2/PGE_2_ pathway. We also compared the TMSCs primed with either IFN-γ or TNF-α to those pretreated with both cytokines simultaneously and found that TMSCs primed by TNF-α exhibited the higher potency of PGE_2_ production, comparable to the co-pretreatment group, than those pre-treated with IFN-γ. Our findings suggest that single TNF-α priming may be sufficient to advance immunosuppressive effects by TMSCs.

Although pre-exposure to inflammatory cytokines can be beneficial for enhancing MSCs-mediated immunomodulation, this may result in undesired adverse effects at the same time. A recent study showed that IFN-γ pre-treatment induced the upregulation of human leukocyte antigen (HLA) molecules on MSCs, which leads to an increased immunogenicity and subsequent vulnerability to host defense mechanisms [38]. In the present study, we observed that TMSCs primed with IFN-γ exhibited relatively high immunogenicity and an insignificant or only moderate improvement in immunosuppression compared to those with TNF-α, presumably due to IFN-γ-mediated upregulation of class II HLA molecules on TMSCs. These results indicate that more attention should be paid to possible changes in the properties of MSCs themselves, such as immunogenicity, as well as the desired enhancing effects when licensing the cells with inflammatory signaling inducer molecules like IFN-γ.

Growing evidence has verified the beneficial efficacies and relevant mechanisms of TMSCs in various animal models of inflammatory disorders, including skin inflammation [39], graft-versus-host disease in an allogeneic bone marrow transplantation [40] and autoimmune-mediated chronic colitis [41]. In particular, inflammatory bowel disease (IBD) characterized by T cell-mediated autoimmune pathogenesis is considered as one of the best disease models to assess the immunomodulatory potency of MSCs in vivo setting, and DSS-induced murine colitis is considered an indispensable model due to its simplicity, rapidity, reproducibility and controllability [42]. According to the study by Yu et al. [41], multiple administration of naïve TMSCs significantly ameliorated murine chronic colitis induced by repeated supply and withdrawal of low concentration (1.5%) DSS solution. In this study, we utilized a 3% DSS-induced acute colitis model to achieve typical symptoms within a short period of time and assess the preventive and therapeutic effects of TMSCs more apparently. In our setting, a single systemic injection of naïve TMSCs also showed decent prophylactic potency to some degree, whereas no statistically significant improvement was observed in the therapeutic assessment. However, only TMSCs primed with TNF-α represented remarkable efficacy in both treatment and prevention, which corroborates the need for pre-treatment to achieve an outstanding potency.

To date, several groups have demonstrated the effects and underlying mechanisms of MSCs pre-treated with inflammatory stimulants such as IFN-γ, polyinosinic:polycytidylic acid (Poly(I:C)) and IL-25 [43,44,45,46], and even one case report documented a clinical trial of IFN-γ-primed BM-MSCs in a patient with childhood-onset and multidrug-resistant IBD, but no remarkable clinical benefit was achieved [47]. There have been also a few papers on the effects of TNF-α pre-treatment on the therapeutic efficacy of MSCs against colitis, but most of them focused on the results of canine AT-MSCs administration in a murine colitis model [48], and no sufficient references currently available. In this respect, we present here the prominent enhancing effect of TNF-α priming on TMSCs immunomodulation, which can be useful for the application to MSCs from other sources or the development and optimization of the pretreatment recipe.

Despite the promising results of TNF-α pre-treatment, there are still areas to be revealed. Since tonsils are a novel MSCs source that has emerged relatively recently, it is necessary to collect more evidence on the characteristics of TMSCs depending on donor and other covariates. We did not observe a significant therapeutic efficacy with single TMSCs administration, but this may need to be further validated with more TMSCs obtained from different donors. In the present study, we observed that the immunosuppressive abilities of TMSCs considerably varied among donors, presumably due to the difference in fundamental donor intrinsic factors, age, degree and duration of inflammation. Given that TMSCs are obtained through tonsillectomies, the basic properties of TMSCs and their responsiveness to the ex vivo priming may be determined depending on the in vivo priming state by the host inflammatory microenvironment at the time of the tissue ablation. The COX-2/PGE_2_ axis was represented as the primary signaling pathway responsible for TMSCs immunomodulation in this study, but other potent mediators and mechanisms from the complicated immune network should be explored through further investigations.

In summary, we demonstrate that TMSCs as a promising cell therapy source can maintain beneficial MSCs properties until relatively late passages, and TNF-α pre-treatment markedly enhances the immunosuppressive effects of TMSCs on T cells and macrophages as well as the preventive and therapeutic potency in a murine acute colitis model. Our findings can provide useful insights into the development of MSCs-based therapy and its enhancement.

## Supplementary Materials

Supplementary materials can be found at https://www.mdpi.com/2227-9059/8/12/561/s1.
