# Supplementary material for: TNF-α Priming Elicits Robust Immunomodulatory Potential of Human Tonsil-Derived Mesenchymal Stem Cells to Alleviate Murine Colitis
Source: Biomedicines. 2020 Dec 2;8(12):561. doi: 10.3390/biomedicines8120561 (PMC7760130; doi:10.3390/biomedicines8120561)
Supplement: Supplementary file 1 [file biomedicines-08-00561-s001.zip › biomedicines-1013563-supplementary.docx]

Supplementary information

TNF-α Priming Elicits Robust Immunomodulatory Potential of Human Tonsil-Derived Mesenchymal Stem Cells to Alleviate Murine Colitis

Tae-Hoon Shin ^1,†^, Ji-Su Ahn ^2,3,†^, Su-Jeong Oh ^2,3^, Ye Young Shin ^2,3^, Ji Won Yang ^2,3^,
Min-Jung Kang ^2,3^, Ji Min Kim ^4^, Byung-Joo Lee ^4^, Yoojin Seo ^2,3,^*^,‡^, and Hyung-Sik Kim ^2,3,^*^,‡^

^1^ Translational Stem Cell Biology Branch, National Heart, Lung, and Blood Institute, National Institutes of Health, Bethesda, MD 20892, USA; thshin1125@gmail.com

^2^ Dental and Life Science Institute, Pusan National University, Yangsan 50612, Korea; anjs08@naver.com (J.-S.A.); dhtnwjd26@naver.com (S.-J.O.); bubu3935@naver.com (Y.Y.S.); midnightnyou@naver.com (J.W.Y.); [kkang085@naver.com](mailto:kkang085@naver.com) (M.J.K.)

^3^ Department of Life Science in Dentistry, School of Dentistry, Pusan National University, Yangsan 50612, Korea

^4^ Department of Otorhinolaryngology-Head and Neck Surgery, Biomedical Research Institute, Pusan National University School of Medicine, Pusan National University Hospital, Busan 49241, Korea; ny5thav@hanmail.net (J.M.K); voiceleebj@gmail.com (B.-J.L.)

***** Correspondence: amaicat24@naver.com (Y.S.); hskimcell@pusan.ac.kr (H.-S.K.); Tel.: +82-51-510-8231 (Y.S.); +82-10-5283-0721 (H.-S.K.)

† These authors contributed equally to this work.

‡ Authors share co-corresponding authorship.


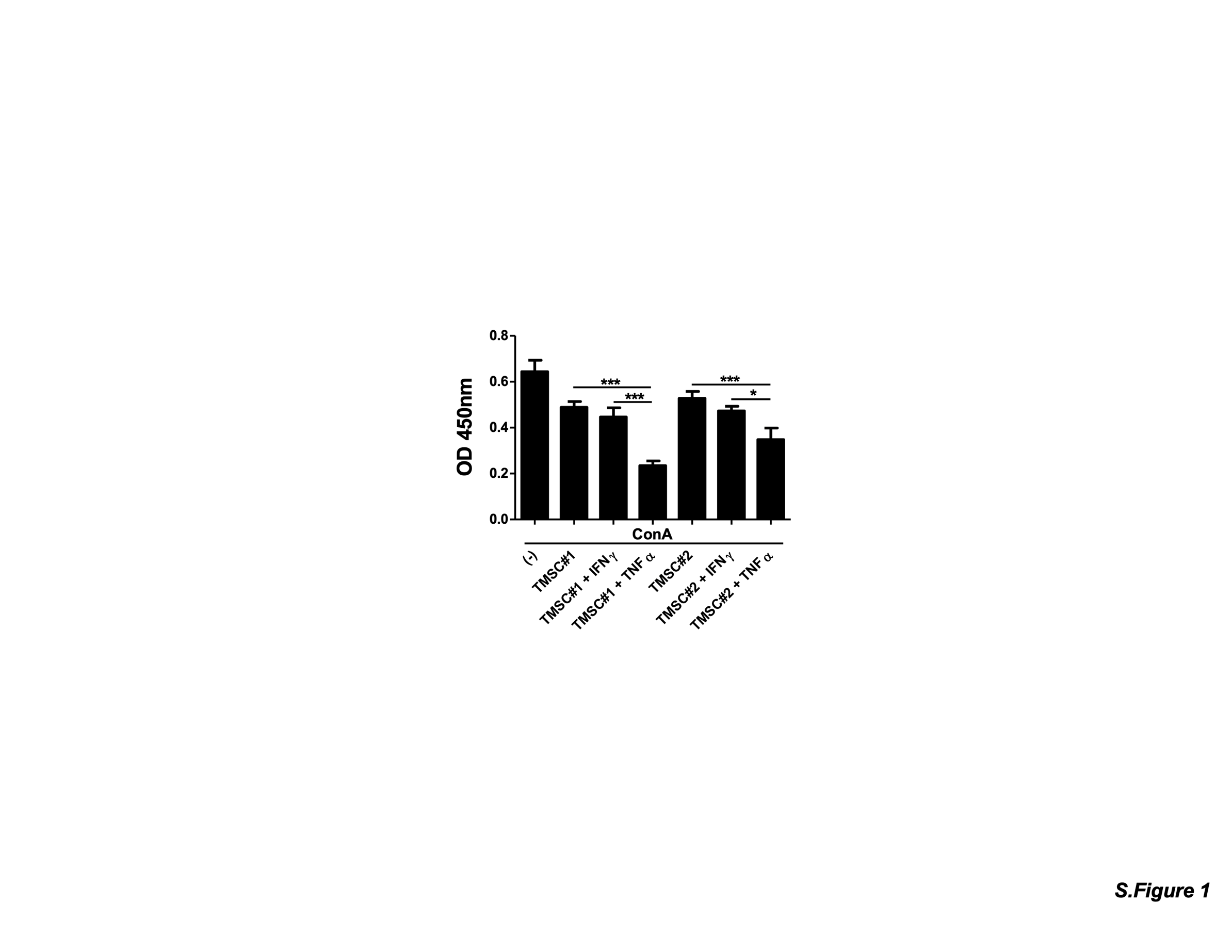


**Figure S1.** Suppressive effects of TNF-α-pretreated TMSCs on the proliferation of mouse splenocyte.
